# Acute Pancreatitis in Mild COVID-19 Infection

**DOI:** 10.7759/cureus.9886

**Published:** 2020-08-20

**Authors:** Seetha Lakshmanan, Amer Malik

**Affiliations:** 1 Internal Medicine, Roger Williams Medical Center, Providence, USA; 2 Gastroenterology, Roger Williams Medical Center, Providence, USA

**Keywords:** pancreatitis, covid-19

## Abstract

The novel coronavirus disease 2019 (COVID-19) caused by severe acute respiratory syndrome coronavirus-2 (SARS-CoV-2) has caused a global health crisis. This virus uses angiotensin converting enzyme 2 (ACE2) receptors to facilitate cellular entry. ACE2 receptors appear to be highly expressed in pancreatic exocrine glands and islet cells, more so than in lung tissues, suggesting that pancreatic injury can occur despite having only mild COVID-19 symptoms. We report a patient with COVID-19 who presented with only gastrointestinal symptoms and discuss the importance of identifying pancreatitis early to improve prognosis.

## Introduction

The novel coronavirus disease 2019 (COVID-19) caused by severe acute respiratory syndrome coronavirus-2 (SARS-CoV-2) has caused a global health crisis, with more than 16.8 million cases worldwide and over 650,000 deaths [1]. Despite learning more about this virus every day, the impact of COVID-19 on the pancreas remains less explored. We report a patient with COVID-19 who presented with pancreatitis in the absence of respiratory symptoms.

## Case presentation

**Figure 1:**
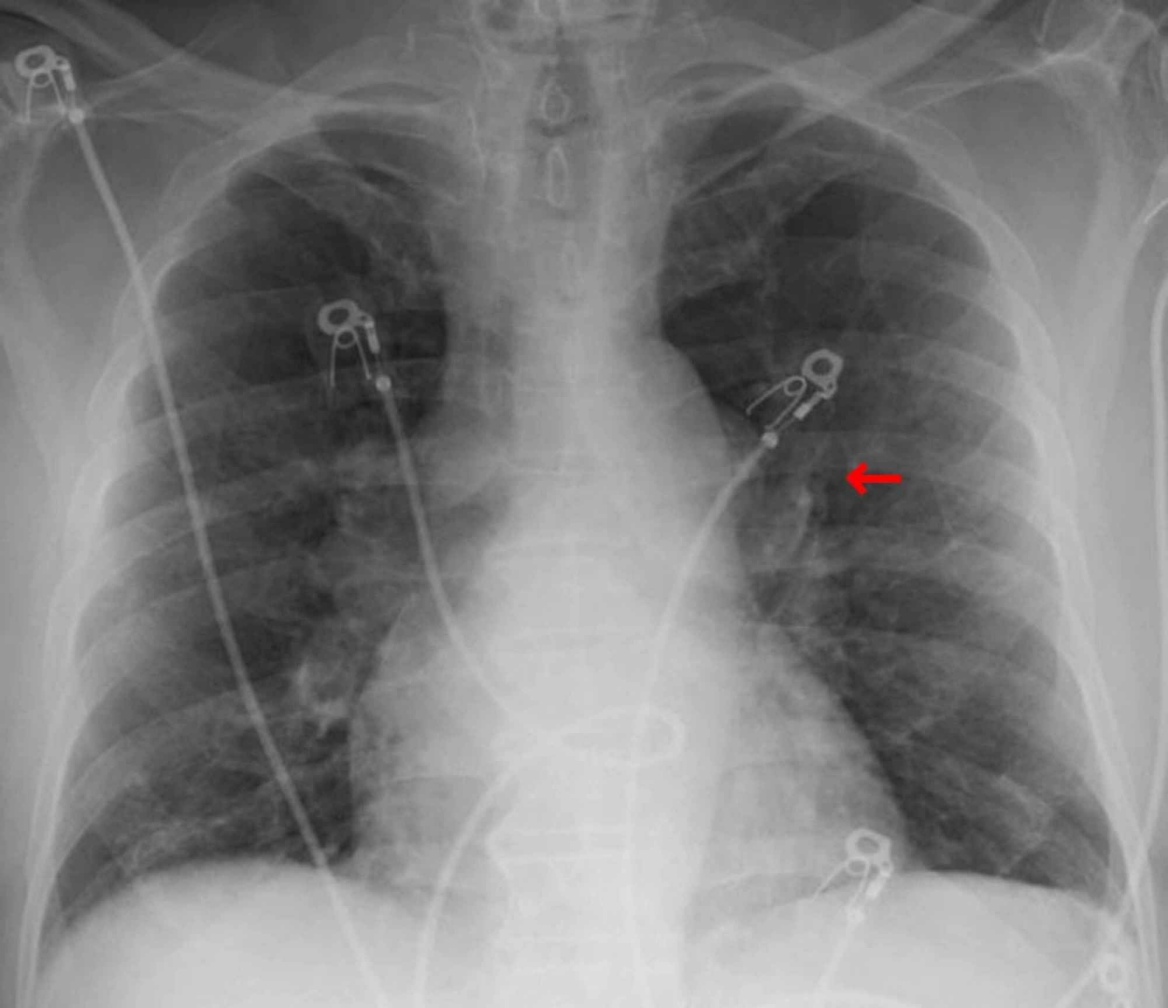
Patient's chest X-ray which appears generally clear with faint opacity in the left perihilar region (red arrow).

The patient was a 68-year-old male nursing home resident, with a past medical history of diabetes mellitus, hypertension, and stage IV chronic kidney disease. He denied any history of tobacco, alcohol, or substance use. After an outbreak of COVID-19 at his residence, a routine polymerase chain reaction (PCR) test was done, which confirmed COVID-19 infection two days prior to presenting to the emergency department (ED). The patient complained of loss of appetite and nausea for one week while denying any respiratory symptoms. In the ED, he was afebrile and saturating 97% on room air. On examination, he appeared dehydrated and lethargic, while his lung sounds were clear and his abdomen was soft and non-tender. Laboratory workup on admission revealed white blood cell (WBC) 8900 ug/L without lymphopenia, sodium 136 mmol/L, blood urea nitrogen (BUN) 77 mg/dl, creatinine 6.6 mg/dl, glucose 46 mg/dl, procalcitonin 6.04 ng/ml, C-reactive protein (CRP) 158 mg/L, aspartate aminotransferase (AST) 27 U/L, alanine aminotransferase (ALT) 20 U/L, alkaline phosphatase (ALP) 123 U/L and total bilirubin of 0.5 mg/dl. The chest radiograph showed a faint patchy opacity in the left perihilar region (Figure 1). He was admitted for acute on chronic kidney injury and hypoglycemia due to poor oral intake. He was also empirically covered with antibiotics for possible superimposed bacterial pneumonia.

**Figure 2:**
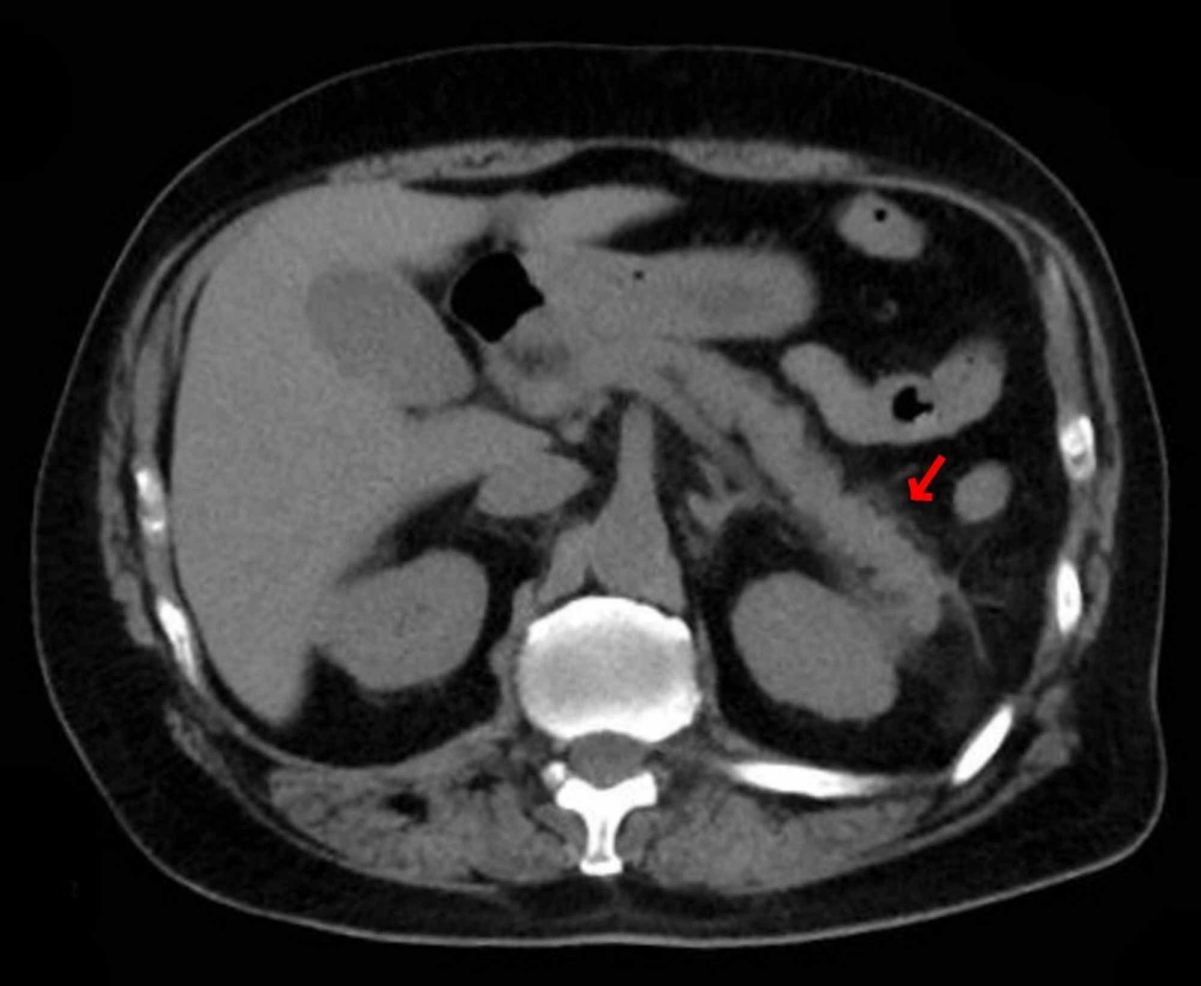
CT abdomen without contrast showing peripancreatic fat stranding greatest around the tail (red arrow). Gallbladder appeared normal without wall thickening or surrounding inflammatory changes and the common bile duct is not dilated.

Over the next few days, the patient's kidney function improved with hydration. He had persistent nausea, vomiting, and anorexia, but no abdominal pain. A non-contrast abdominal computed tomography (CT) scan revealed peripancreatic fat stranding, greatest around the tail, with mild duodenal wall thickening and adjacent fat stranding, likely from pancreatitis. The gallbladder appeared normal, without wall thickening or surrounding inflammatory changes, and the common bile duct was not dilated (Figure 2). Amylase and lipase levels were elevated at 1030 U/L and 2035 U/L, respectively, but the triglyceride and calcium levels were normal. The patient was kept on bowel rest, and given continuous intravenous fluids and antiemetics. Throughout the admission, he did not have any respiratory signs or symptoms, nor did he require oxygen supplementation. Repeat chest radiograph did not show any focal consolidations. The patient's appetite eventually improved, and his diet was advanced. He was discharged seven days later back to his nursing home with a follow-up appointment.

## Discussion

SARS-CoV-2, which is about 80% genetically similar to SARS-CoV, enters cells via binding of a viral spike protein to angiotensin converting enzyme 2 (ACE2) receptors, facilitating cellular entry. This binding downregulates ACE2 receptors in certain tissues, thereby decreasing ACE2 expression and exacerbating tissue injury. ACE2 receptors appear to be highly expressed in pancreatic exocrine glands and islet cells, more so than in lung tissues. While the literature shows that ACE2 receptor downregulation occurs in the lungs, it remains to be said if the same process occurs in the pancreas or the clinical implications that it may have [2-5]. Although there have been reports of pancreatic injury in SARS-CoV resulting in hyperglycemia from islet cell damage, none were known to have developed pancreatitis [5].

Roughly 10% of acute pancreatitis cases are from an infectious etiology. Since the onset of the COVID-19 pandemic, there have been few patients reported with COVID-19-related acute pancreatitis, each of whom presented with varying symptoms and were diagnosed with pancreatitis at different stages of their respective hospitalizations [6-8]. Three of them were admitted to the intensive care unit for multi-organ failure, while two were intubated due to acute hypoxic respiratory failure. Our patient was the first to have developed acute pancreatitis without any respiratory complications throughout the course of infection.

There are retrospective studies reporting pancreatic injury related to COVID-19, but they do not fit all the criteria for diagnosing acute pancreatitis. The revised Atlanta classification for acute pancreatitis requires two of the following: (1) acute onset of severe epigastric pain, often radiating to the back; (2) serum lipase or amylase levels greater than three times the upper limit normal; and (3) characteristic findings of acute pancreatitis on abdominal imaging (contrast-enhanced CT, ultrasound, magnetic resonance imaging). The severity may then be categorized as mild (absence of organ failure or complications), moderately severe (transient organ failure with or without local or systemic complications), and severe (organ failure lasting longer than 48 hours) [9]. Our patient had a moderate acute kidney injury while hospitalized, but did not develop any organ failure. Thus, his course can be classified as mild acute pancreatitis.

An early study by Wang et al. reported that nine out of 52 (17%) patients with COVID-19 experienced pancreatic injury characterized by any abnormality in the pancreatic enzymes, but did not consider clinical signs and symptoms or imaging studies. Without additional evidence, mild rises in enzyme levels can be attributed to other factors, including poor excretion due to acute renal failure or increased secretion of amylase by the salivary glands and lungs, which are also affected by COVID-19. Another cohort study later highlighted that patients with severe cases of COVID-19 (17.9%) compared to mild cases (1.85%) were more likely to have elevated amylase and lipase levels. However, only 7.5% of patients had concurrent pancreatic injury confirmed by imaging studies [5].

## Conclusions

This case highlights that even in mild cases of COVID-19, clinicians should be wary of pancreatitis, especially if the patient develops gastrointestinal signs and symptoms. Prompt diagnosis is essential for improving prognosis and quickening recovery of these patients. Further research is necessary to investigate the incidence and severity of pancreatic inflammation and its correlation to respiratory symptoms.
